# Do social accountability approaches work? A review of the literature from selected low- and middle-income countries in the WHO South-East Asia region

**DOI:** 10.1093/heapol/czaa107

**Published:** 2020-11-09

**Authors:** Nahitun Naher, Dina Balabanova, Eleanor Hutchinson, Robert Marten, Roksana Hoque, Samiun Nazrin Bente Kamal Tune, Bushra Zarin Islam, Syed Masud Ahmed

**Affiliations:** c1 Centre of Excellence for Health Systems and Universal Health Coverage (CoE-HS&UHC), BRAC James P. Grant School of Public Health, BRAC University, 5th Floor (Level-6), ICDDR,B Building, 68 Shahid Tajuddin Ahmed Sharani, Mohakhali, Dhaka 1212, Bangladesh; c2 Department of Global Health and Development, London School of Hygiene and Tropical Medicine (LSHTM), 15-17 Tavistock Place, London, WC1H 9SH, UK; c3 Alliance for Health Policy and Systems Research, Science Division, World Health Organization, avenue Appia 20, 1211, Geneva 27, Switzerland

**Keywords:** Health systems, social accountability approaches, transparency, accountability, community participation, governance

## Abstract

Governance failures undermine efforts to achieve universal health coverage and improve health in low- and middle-income countries by decreasing efficiency and equity. Punitive measures to improve governance are largely ineffective. Social accountability strategies are perceived to enhance transparency and accountability through bottom-up approaches, but their effectiveness has not been explored comprehensively in the health systems of low- and middle-income countries in south and Southeast Asia where these strategies have been promoted. We conducted a narrative literature review to explore innovative social accountability approaches in Bangladesh, Bhutan, India, Indonesia, the Maldives, Myanmar and Nepal spanning the period 2007–August 2017, searching PubMed, Scopus and Google Scholar. To augment this, we also performed additional PubMed and Google Scholar searches (September 2017–December 2019) to identify recent papers, resulting in 38 documents (24 peer-reviewed articles and 14 grey sources), which we reviewed. Findings were analysed using framework analysis and categorized into three major themes: transparency/governance (eight), accountability (11) and community participation (five) papers. The majority of the reviewed approaches were implemented in Bangladesh, India and Nepal. The interventions differed on context (geographical to social), range (boarder reform to specific approaches), actors (public to private) and levels (community-specific to system level). The initiatives were associated with a variety of positive outcomes (e.g. improved monitoring, resource mobilization, service provision plus as a bridge between the engaged community and the health system), yet the evidence is inconclusive as to the extent that these influence health outcomes and access to health care. The review shows that there is no common blueprint which makes accountability mechanisms viable and effective; the effectiveness of these initiatives depended largely on context, capacity, information, spectrum of actor involvement, independence from power agendas and leadership. Major challenges that undermined effective implementation include lack of capacity, poor commitment and design and insufficient community participation.

**KEY MESSAGES**

- Governance failures undermine efforts to achieve universal health coverage and improve health in low- and middle-income countries by decreasing efficiency and equity.
- A variety of context-specific social accountability interventions have been tried in Bangladesh, Bhutan, India, Indonesia, the Maldives, Myanmar and Nepal. The evidence suggests that such properly designed and implemented interventions enhance and supplement existing accountability mechanisms.

## Introduction

Improving health systems’ responsiveness, quality and efficiency remains an ongoing challenge in many low- and middle-income countries (LMICs) (Panda and Thakur, 2016), and enhancing the quality of governance and accountability is increasingly critical in achieving this. Although there are debates on how these objectives should be sequenced, there is growing recognition that achieving them requires managerial ‘good governance’ models and bottom-up social accountability approaches involving a variety of community actors. According to the World Bank’s ‘long and short route’ framework of accountability, with the long route, citizens influence policymakers and policymakers in turn influence service providers. When this long route breaks down, there are fewer opportunities to ensure service provision is accessible and equitable. Given the lengthy time that the long route of accountability takes in many settings, it is expected that service outcomes could be better improved by strengthening the short route through increasing citizens’ power over providers (World Bank Group, 2004).

Social accountability refers to an approach that focuses on civic engagement, i.e. ordinary citizens and/or civil society organizations participating directly or indirectly in policy processes to ensure that their concerns are taken into account and services are responsive to their needs (Carmen *et al.*, 2004). Accountability in this context is the willingness of politicians to justify their actions and to accept electoral, legal or administrative penalties as appropriate. Two of the key aspects of social accountability are answerability (the right to receive relevant information and explanation for actions) and enforceability (the right to impose sanctions if the information or rationale are deemed inappropriate) (George, 2003; Ackerman, 2004). Answerability ensures the compulsion of policymakers or service providers to meet performance goals, while enforceability requires actions with penalty following failure to comply (Bruen *et al.*, 2014). The voice is the instrument of accountability between citizens and politicians, with a range of measures through which citizens express their preferences and influence politicians. Improved accountability requires citizens to have a voice when it comes to locally elected leaders who can hold public servants to account (Lewis, 2006). However, this is not sufficient for accountability; it may lead to answerability, but it does not necessarily lead to enforceability (World Bank Group, 2004). Initiatives seeking to promote meaningful participation and accountability are often considered an important element to improve health system performance (Lewis, 2006; Vian, 2007; Ringold *et al.*, 2012).

Social accountability should be considered as a multi-pronged process that utilizes multiple tactics, encourages collective action and voice alongside governmental reforms that bolster public sector responsiveness and facilitates outcomes and impacts that are more promising (Boydell, 2018). The concept reflects complex interactions between different stakeholders who have varying degrees of interest and power at different points in the service delivery process (Brinkerhoff, 2004; Ringold *et al.*, 2012; Joshi, 2013).

A range of social accountability mechanisms, e.g. participation, watchdog organizations, scorecards and public hearings, have been implemented in South/Southeast Asian countries, with different forms often implemented as packages (Boydell and Kissbury, 2014). Their aims are diverse, including improving access to and quality of care, empowering community stakeholders and improving service efficiency. Thus, the emerging question that we seek to address in this review is: what are the social accountability initiatives that have been implemented, how did they function and what made them successful or not? This review seeks to address this critical gap in the literature and to inform the design of innovative, bottom-up community-based approaches to improve health sector governance.

## Methods

### Scope and objective of the review

This literature review presented here is a subset of a larger review on corruption in Bangladesh, which highlighted a range of participatory and social accountability mechanisms. This review sought to synthesize the evidence specifically on social accountability in selected countries in Southeast Asia, and its implication for strengthening health sector governance, addressing corruption and improving health system performance through citizens’ engagement in Bangladesh, Bhutan, India, Indonesia, the Maldives, Myanmar and Nepal.

### Case definitions

To answer our question, we developed a literature review protocol using a search strategy identifying the scope and methods for the review (data sources, key search terms and eligibility criteria). The definitions of key concepts used are described in Table 1.

**Table 1:** Definition of key concepts used

Key concept	Definition	Source
Corruption	Corruption is the abuse of entrusted power for private gain. It can be classified as grand, petty and political, depending on the amounts of money lost and the sector where it occurs	Transparency International (2009)
Community participation	Involvement of people in a community in projects to solve their own problem	World Bank Group (2012)
Citizen’s Charter	Citizen’s Charters are part of the new public management approach and are initiated to encourage service providers to be responsive and to inform citizens about service entitlements, standards and rights	Shamra (2012)
Decentralization	Socio-political process of power-sharing arrangements between central government and local authorities in planning, management and decision-making	Regmi *et al.* (2010b)
Governance	The manner in which power is exercised in the management of a country’s economic and social resources for development	World Bank (1992)
Good governance	Exercise of power through institutions to steer society for the public good	Huss *et al.* (2011)
Public hearing	Formal meetings at the community level where citizens express their grievances on matters of public interest to public officials who try to address their grievances	Ahmed (2016)
Social accountability	An approach towards building accountability that relies on civic engagement, i.e. in which it is ordinary citizens and/or civil society organizations that participate directly or indirectly in exacting accountability	Carmen *et al.* (2004)
Social audit	A means of independently monitoring or evaluating the performance of an organization in attaining its social goal	World Bank Group (2012)
Transparency	A characteristic of governments, companies, organizations and individuals of being open in the clear disclosure of information, rules, plans, processes and actions	Transparency International (2009)

We searched English-language literature papers between January 2007 and August 2017 in the initial phase and later extended the search from September 2017 to December 2019. The search focused on Bangladesh, Bhutan, India, Indonesia, Myanmar, the Maldives and Nepal, which is where the majority of the social accountability initiatives have been implemented and there is a critical mass of studies, enabling us to draw conclusions. For published journal articles (peer-reviewed), we searched electronic databases PubMed, Scopus and Google Scholar. We used the search terms ‘Corruption’, ‘Informal payment’, ‘Anti-corruption’, ‘Governance’, ‘Good governance’, ‘Accountability’, ‘Social accountability’, ‘Community’, ‘Healthcare provider’, ‘Health service’ and ‘Healthcare facility’, and combined these with the countries’ names (Table 2 key search terms). The search terms were used in combination with the Boolean operator ‘AND’. In addition, we searched the Internet to identify relevant grey literature. We also searched databases of relevant international organizations, e.g. the World Health Organization, Transparency International (TI) and the World Bank Group, to identify relevant papers and reports (Table 3 search protocol).

**Table 2:** Keywords for searching electronic databases

Corruption (combined with ‘AND’) (a)^a^	Governance/accountability (combined with ‘AND’) (b)^a^	Health sector (combined with ‘AND’) (c)^a^	Geographic location (combined with AND’) (d)^a^
Corruption Informal payment Anti-corruption Anti-corruption strategies	Governance Good governance Accountability Social accountability	Healthcare provider Healthcare service Health facilities	WHO SEAR LMICS (Selected Countries) Bangladesh Bhutan India Indonesia Myanmar Maldives Nepal

a a, b, c and d groups were combined with Boolean operator ‘AND’.

**Table 3:** Search protocol

Scope	Synthesize evidence on good governance and social accountability approaches	
Search strategy	Inclusion criteria	Peer-reviewed journal articles, reports, programme documents, blogs and other grey materials; websites of relevant organizations and institutions
		Language: English
	Exclusion criteria	Countries other than WHO SEAR LMICs, beyond timeframe, documents in other languages, documents’ full text unavailable
	Timeframe	January 2007–August 2017 (original search) September 2017–December 2019 (extended search)
Search terms	Corruption, Governance, Social Accountability, Health Sector, Southeast Asia	
Data source	Electronic database	PubMed, Scopus, Google Scholar
	Grey literature	Google
	Institutional websites	WHO, World Bank, TI

### Search retrieval and analysis of studies

Two researchers screened the list of articles independently. Titles and abstracts of the articles were read to determine their relevance to the topic. After excluding duplicate citations, we excluded non-peer-reviewed journal articles, articles not in the English language and those published before January 2007. We further excluded editorials, proposals and protocols that contained no empirical findings. For papers meeting the inclusion criteria, the researchers retrieved the full text for further assessment. Any disagreement was resolved through discussion with the project lead. We used framework analysis as an analytical strategy, to identify key themes and divergent findings within the literature, explore their characteristics and synthesize data (Gale *et al.*, 2013). A sample of sources was read and re-read by the study team members (NN, RH, SMA) independently to develop the initial coding matrix of themes. This was discussed, refined and agreed before the remainder of the sources were reviewed and analysed using the agreed coding matrix. Researchers independently extracted data from included studies using the matrix, which helped to develop a working analytic framework. Differences in data extraction were resolved by discussion with the broader team, to ensure quality. After extraction and tabulation, we categorized the data into themes (transparency, accountability and community participation) for analysis. Once data had been extracted and classified according to themes, the researchers read and re-read the papers to identify and confirm the classification. The categories were grouped under three major sub-themes: ‘transparency’, ‘accountability’ and ‘community participation’. This approach facilitated the analysis; key findings were summarized from each matrix column, identifying the commonalities and differences.

## Results

**Figure 1:**
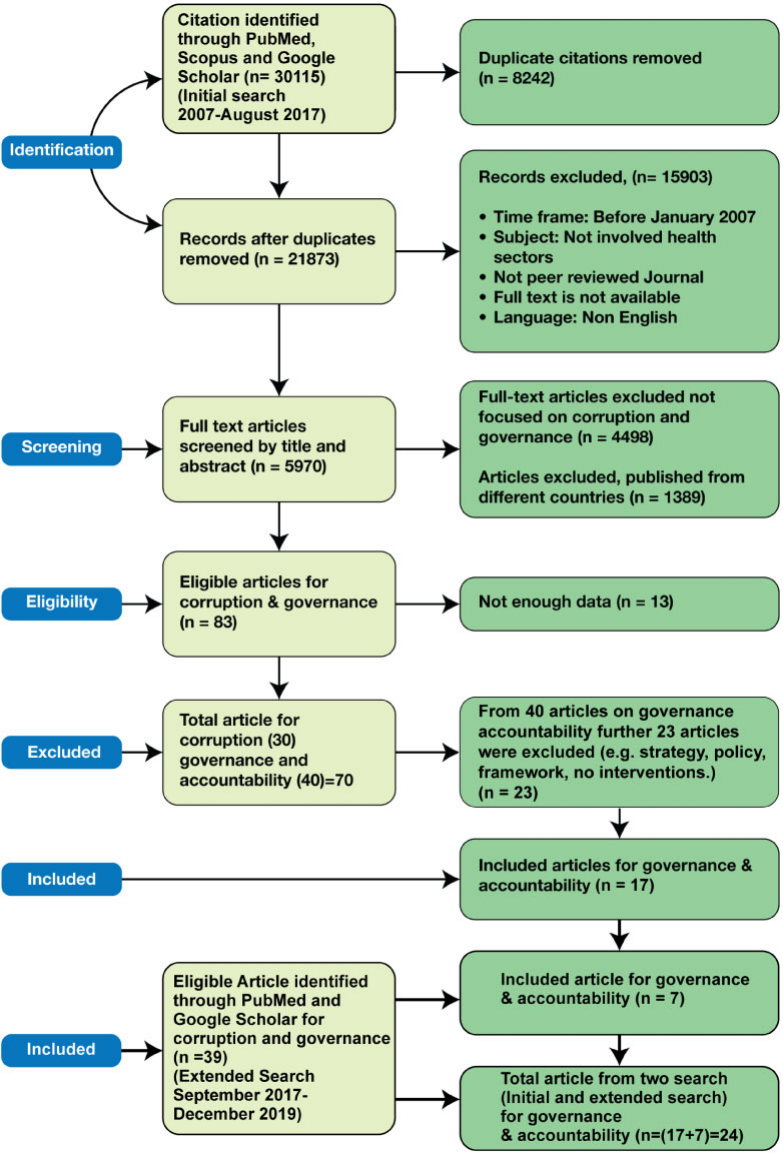
PRISMA diagram

From the initial search, we identified a total of 30 115 articles from the three electronic databases. The preferred reporting items for systematic reviews and meta-analysis (PRISMA) flow are presented in Figure 1. After removing 8242 duplicates, 21 873 articles were screened, and a further 15 903 articles were excluded as they were not in English, were published before 2007 or full text was not available. From the remaining 5970 articles, a further 4498 articles were excluded as they were not focused on social accountability issues. A further 1389 articles were excluded as they did not refer to the selected countries. Finally, 83 articles were read. Of these, 13 were excluded as they did not include sufficient data for analysis and 30 were excluded as they focused on corruption issues broadly. Out of the remaining 40 social accountability and governance-related papers, we further excluded 23 that discussed possible policies and strategies rather than specific interventions and also those that did not include any outcomes. Out of these, we included 17 papers, of which six were on transparency and governance issues, seven on accountability mechanisms and four on community participation approaches. Apart from the journal articles, an additional 15 documents were also selected that included project and programme reports of national and international organizations, from which finally nine were reviewed. A total of 26 documents (17 journal articles and nine grey literature sources) were reviewed for the paper from the initial search (Figure 1 PRISMA diagram).

The updated PubMed and Google Scholar searches identified an additional 39 documents, of which 12 (seven journal articles and five grey literature sources) were identified and reviewed for this paper (Table 4). Thus, a total of 38 documents (24 journal articles and 14 grey literature sources) were included in this paper from the two search phases.

**Table 4:** Description of documents from two-phase search

Type and theme of documents reviewed	Number of documents reviewed		Reference of documents reviewed	
Systematic reviews	From initial search	From extended search	From initial search	From extended search
Governance and transparency	3	1	Houque and Ahsan (2016), Dieleman *et al.* (2011), Garimella and Sheikh (2016)	Juwita (2018) (August)
Accountability	3	2	Regmi *et al*. (2010a), Cleary *et al.* (2013), Prasuna and Kumar (2016)	Kumar (2019), Shohag (2018)
Community participation	1	1	Kok *et al.* (2015)	Blair (2018)
Total review based articles = 11				
Quantitative studies	From initial search	From extended search	From initial search	From extended search
Governance and transparency	1		Panda *et al.* (2016)	
Accountability	2		Khan *et al*. (2013), Gurung and Tuladhar (2013)	
Community participation	2		Wangmo *et al.* (2016), Bhuiya *et al.* (2016)	
Total quantitative study based articles = 5				
Qualitative studies	From initial search	From extended search	From initial search	From extended search
Governance and transparency	2		Huss *et al.* (2011), Sharma (2012)	
Accountability	2	3	Devkota *et al*. (2013), Regmi *et al.* (2010b)	Hamal e*t al*. (2019), Gurung *et al*. (2019), Dhungana (2019)
Community participation	1		Mishra (2014)	
Total qualitative study based articles = 8				
Total number of journal articles reviewed (17 from initial search + 7 from extended search) = 24				
Additional documents reviewed	From initial search	From extended search	From initial search	From extended search
National/international organizations project reports	6		GIZ (2014), GTZ (2009), 3MDG (2012), 3MDG (2016), Asia Pacific Network (2011), Ahmed (2016)	
Case studies	1		COPASAH (2015)	
Working paper	1	3	PTF (2012)	Naher *et al.* (2018), Khan *et al.* (2019), Bhattacharya *et al.* (2018)
Blog	1		SHARE (2016)	
Book		2		Juwita (2018), Rosenbloom (2017)

Total number of additional documents reviewed (nine from initial search + five from extended search) = 14.

Grand total of documents reviewed for this paper (24 journal articles + 14 additional documents) = 38.

### Study design and country of origins

Studies were selected irrespective of their study design, and included a variety of designs. From the initial search; out of 17 journal articles, seven were reviews, five were quantitative studies, four were qualitative studies and one was a case study. Out of the nine grey literature sources, there were six project reports, one case study, one working paper and one blog (Table 4). Out of the 26 documents from the initial search, six each were from Bangladesh, India and Nepal. Three were from Myanmar, with one each from Bhutan and Indonesia and three from LMICs (Table 5).

**Table 5:** Social accountability approaches tested across different countries in WHO Southeast Asia region

Social accountability elements	Social accountability tools tested	Countries who tested the tools
Transparency	Citizen’s Charter	Bangladesh, India, Nepal
	Online platform	Bangladesh, India
	Advice and information desk	Bangladesh, India
	Awareness campaign	Bangladesh, India, Indonesia
Accountability	Social audit	Bangladesh, India, Nepal
	Decentralization	India, Maldives
	Office of Ombudsman (e.g. Lokpal)	India
	Hospital management committee	Bangladesh
	Citizen committee/Monitoring group	Bangladesh, India
	Participatory complaints survey	Indonesia
	Community score cards	India, Maldives, Myanmar, Nepal
	Citizen report cards	India, Maldives
	Complaint box	Indonesia
Community participation	Public hearing, public dialogue, public theatre and campaign	Bangladesh, India
	Patient welfare committee	India
	Village health development committee	Nepal
	School programme	India
	Community of concerned citizens/action group	Bangladesh, Bhutan, India
	Women’s group (NariDal)	Bangladesh

From the extended search, out of seven journal articles, four were reviews and three were qualitative studies. Out of the five grey literature documents, three were working papers and two were book chapters (Table 4). Out of these 12 newly reviewed documents from the extended search, two were from Indonesia, three each were from India and Nepal and four were from Bangladesh.

Out of the 24 journal articles identified during the two-phase search, 11 were reviews, five were quantitative studies and eight were qualitative studies. Out of the 14 grey literature sources, one was a case study, four were working papers, one was a blog, two were book chapters and six were project reports (Table 4).

From the 38 documents reviewed through the updated search, 10 were from Bangladesh, nine each were from India and Nepal, three each were from Myanmar and Indonesia, one was from Bhutan and the remaining three were from mixed LMICs.

### Scope and challenges of the approaches practised

The social accountability strategies and interventions implemented to enhance transparency, accountability and community participation varied widely (Table 6). Despite some positive outcomes and process features, most of these approaches had important limitations as well.

**Table 6:** Summarized key findings from reviewed documents

Articles	Authors	Context	Scope	Intervention example	Evidence on impact
**Good governance**					
Good governance and corruption in the health sector: lessons from the Karnataka experience.	Huss *et al.* (2011)	Strengthening good governance and preventing corruption in health care are universal challenges.	To evaluate KLA experience.	The Karnataka Lokayukta (KLA), a public complaints agency in Karnataka state (India), was created in 1986	Played a prominent role controlling systemic corruption only after a change of leadership in 2001 with a new Lokayukta (ombudsman) and Vigilance Director for Health (VDH).
Improving the implementation of health workforce policies through governance: a review of case studies	Dieleman et al., 2011	Responsible governance is crucial to national development and a catalyst for achieving the Millennium Development Goals.	How governance issues have influenced HRH policy development and to identify governance strategies that have been used, successfully or not, to improve HRH policy implementation in low- and middle-income countries (LMIC).	Evaluate a governance-related intervention at country or district level in LMIC.	The dimension ‘performance’ covered several elements at the core of governance of HRH, decentralization being particularly prominent. Although improved equity and/or equality was, in a number of interventions, a goal, inclusiveness in policy development and fairness and transparency in policy implementation did often not seem adequate to guarantee the corresponding desirable health workforce scenario.
Health sector corruption as the archenemy of universal health coverage in Indonesia	Ratna Juwita (2018)	Health sector corruption is a direct threat towards achieving universal health coverage in Indonesia.	Health sector corruption is exemplified in the analysis of several national case laws.	Three Indonesian legal cases of health sector corruption were selected to analysis the reality of health sector corruption and its detrimental effect to right health.	Health sector corruption has directly reduced fund for universal health coverage in Indonesia. A people centred right based approach is needed to imply.
Good governance and anti- corruption: Responsibility to protect universal health coverage in Indonesia	Ratna Juwita (2018)	The establishment of universal health care marks a new momentumfor the progressive realization of the right to health in Indonesia.	The problem of corruption in health sector endangers the sustainability ofeffective and quality health care, therefore, Indonesia established an anti-fraud system to protect the universal health insurance fund.	Analyze the current anti-fraud system in universalhealth insurance through the lens of international law and principles of good governance. The sociolegal approach is chosen to study therelationship between the State party obligations to international lawand the implementation of concerning universalhealth care and anti-corruption in the designated anti-fraud system.	Good governance principles are essential in designing an effectiveanti-fraud system due to the correlation between human rights and anti-corruption that both areas emphasize good governance principles as guiding principles for the realization of human rights and the making of potent anti-corruption strategy.

**Local governance**					

Health worker posting and transfer at primary level in Tamil Nadu: Governance of a complex health system function	Garimella and Sheikh (2016)	Posting and transfer (PT) of health personnel – is a contested domain, driven by varied expressions of private and public interest throughout the chain of implementation.	To investigate policymaking for PT in the government health sector and implementation of policies as experienced by different health system actors.	Case study of a PT reform policy at primary health care level in Tamil Nadu State, to understand how different groups of health systems actors experience posting and transfer.	The imperative of enforcing rules may need to be complemented with bottom-up policy approaches, including treating PT not merely as system dysfunction, but also as a potential instrument of governance innovations, procedural justice and the accountability of health services to communities they seek to serve.
Local self-governance in health-a study of it’s functioning in Odisha, India.	Panda *et al.* (2016)	Local decision making is linked to several service quality improvement parameters.	RogiKalyanSamitis (RKS) at peripheral decision making health units (DMHU) are working to ensure accountability and transparency in governance, improve quality of services, and facilitate local responsiveness.	Perception of RKS members about their roles, involvement and practices with respect to local decision making and management of DMHUs.	About 87 % respondents were satisfied with their role in the local governance of the health units.
The Prospect of Accountability in Local Governance in Nepal	Dhungana (2019)	Government accountability is intrinsic to democracies, as citizens can choose public officials through their popular vote and accordingly exercise some control and oversight over the officials.	Accountability in local government requires attention not only to laws, but also the practices of civic interaction and the willingness of elected officials and citizens in these engagements.	Examines how to confront this challenge of holding the governments to account, by looking into local governance in Nepal, where citizens have limited knowledge of the government decisions, activities, procedures followed, and their outcomes.	There is a need to foster greater civic demands on accountability and foster measures for deliberation at the municipal level on a more regular basis. Overall, local government accountability should be envisioned as a work-in-progress pursuit and should be coupled with systems of local planning and implementation and vitalization of local democracy.

**Citizen charter**					

An Evaluation of a citizen‘s charter in local government: a case study of Chandigarh, India.	Sharma (2012)	The Citizen’s Charter, as one of the strategies of New Public Management, aims at providing quality services within a particular timeframe.	It has been introduced in local government with the view of enhancing the excellence of public service deliverance in a responsive, transparent and accountable manner, which in turn aims at increasing the level of satisfaction.	Studying the Citizen’s Charter being formulated by the Municipal Corporation Chandigarh, its implementation and effectiveness from point of view of the agency and as well from the citizens	Intervention was a sheer failure and mere copying of the document for sake of procedural formalities. The reason behind this failure is lack of political will, failure of advertising and poor participation of the people.
Citizen's charter and implementation failure: performance and local councils in Bangladesh.	Huque and Ahsan (2016)	Citizen's charters are tools of empowerment and governments of developing countries are increasingly moving towards adopting them	An analysis of the implementation of charters reveals useful insight on the challenges faced by developing countries in such initiatives	Implementation in local councils	A top-down approach adopted in formulating the charter further contributed to the ineffectiveness of the charter. Citizens found it difficult to access services and were dissatisfied with their quality.
Corruption in the Service Sectors: Revelation of a Pragmatic Explanation in Context of Bangladesh	Shohag (2018)	Corruption is a burning issue of governance. Corruption is not only prevalent in political arena but also in administrative and judicial arena of the country.	Different corruption related activities have been ensued in Bangladesh at the course of many times. This research is based on corruption in the service sectors in Bangladesh.	It has scrutinized the overall scenario of corruption and irregularities in the service sectors in Bangladesh and finally it has examined the scenario and experiences of corruption and irregularities in the service sectors in Bangladesh.	To control service sector corruption appropriate monitoring and oversight mechanisms must be in place in each institution, transparency and integrity has to be ensured in the public procurement both with respect to large procurements, appointments, promotions postings and transfers in all institution serving public interest must be based on merit, expertise and experience an citizen's Charter has to be introduced, enforced.

**Decentralization**					

Health Governance at Local Level from Human Resource for Health Perspectives: the Case of Nepal	Devkota *et al.* (2013)	Evidence about effects of good governance in Human Resources for Health (HRH) is scant in Nepal.	The study aimed to explore the situation of health governance at the local level and suggest measures to address the HRH challenges.	Ninety health facilities.	Only 49 of the health facilities have properly displayed signboard, 42 citizen charter, 36 free health services and Information. Seventy two out of 90 health facilities have not displayed social audit reports and 80 (89%) of the health facilities have not maintained complaint box. The initiative of decentralized human resource management increased ownership at the local level. Nepotism and power exercise was frequently reported as a hindrance.
Decentralization and district health services in Nepal: understanding the views of service users and service providers	Regmi *et al.* (2010a)	Within the decentralization framework of Government, the Ministry of Health (MoH) Nepal initiated the decentralization of primary care services closer to citizens.	Examine and understand the effect of decentralization at the district health service from the perspectives of service users and providers.	District health facilities.	Decentralization was positively associated with increased service access and utilization and improved service delivery. Problems described included three main areas: functions, functionaries and funding.
Understanding the effect of decentralization on health services: the Nepalese experience	Regmi *et al.* (2010b)	Despite enormous progress in health globally, primary healthcare services in many developing countries are facing different challenges.	Assess the effect of decentralization on health services, and to draw general lessons which might help to develop appropriate strategies to improve health services in Nepal.	Decentralization in many countries, including Nepal, suggests a new form of service delivery.	Decentralization in many cases has improved access to, utilisation of, and management of health services. The effects on other performance dimensions such as policy, equity, quality and service effectiveness are poorly investigated.
Decentralized Governance, Corruption and Anti-corruption Measures: An Enquiry in Bangladesh Experience	Bhattacharya *et al.* (2018)	Poor governance system characterized by fractured democratic polity, low level of devolution of power and prevalence of widespread corruption have been considered to be some of the critical structural constraints holding back Bangladesh from the path of inclusive and sustainable development.	Proper implementation of the existing legal provisions and regulations, strengthening the capacity of the local government institutions, improving the local public service delivery and embedding anti-corruption measures and movement have been considered to be the critical antidotes to curb corruption in the country.	Focuses on decentralized governance, corruption and anti-corruption measures intending to improve understanding of the relationships among these concepts in the context of Bangladesh.	There are mixed effects of decentralization on corruption and that the types, dynamics of corruption and impact of different anti-corruption approaches may vary in different decentralized settings. Effective decentralization of authority is yet to be established at the local level.Conflicting provisions regarding administrative authority affects the effective functioning of the governance of the local level institutions

**Score Card**					

Use of a Balanced Scorecard in strengthening health systems in developing countries: an analysis based on nationally representative Bangladesh Health Facility Survey	Khan *et al.* (2013)	Importance of collecting facility‐based data through regular surveys to supplement the administrative data, especially for developing countries of the world.		Health facility survey.	Score card was reported useful for monitoring quality in a health facility survey done in 80 upazila health complex (sub-district health facilities) in Bangladesh.

**Facility survey**					

Resources, attitudes and culture: an understanding of the factors that influence the functioning of accountability mechanisms in primary health care settings.	Cleary *et al.* (2013)	Accountability mechanisms are governance tools that seek to regulate answerability between the health system and the community (external accountability) and/or between different levels of the health system (bureaucratic accountability).	Examines the factors that influence the functioning of accountability mechanisms and relationships within the district health system.	Draws out the implications for responsiveness to patients and communities.	Bureaucratic accountability mechanisms often constrain the functioning of external accountability mechanisms. It is important to limit the potential negative impacts on responsiveness of new bureaucratic accountability mechanisms.

**Community Participation**					

‘Trust and teamwork matter’: Community health workers' experiences in integrated service delivery in India	Mishra (2014)	A comprehensive and integrated approach to strengthen primary health care has been the major thrust of the National Rural Health Mission (NRHM) that was launched in 2005 to revamp India’s rural public health system.	Though the logic of horizontal and integrated health care to strengthen health systems has long been acknowledged at policy level, empirical evidence on how such integration operates is rare.	NRHM primary health care, India.	It shows that for health workers, the notion of integration goes well beyond a technical lens of mixing different health services. Crucially, they perceive ‘teamwork’ and ‘building trust with the community’ (beyond trust in health services) to be critical components of their practice.
Which intervention design factors influence performance of community health workers in low-and middle-income countries? A systematic review.	Kok *et al.* (2015)	Community health workers (CHWs) are increasingly recognized as an integral component of the health workforce needed to achieve public health goals in low and middle-income countries (LMICs).	Many factors influence CHW performance.		A mix of financial and non-financial incentives, predictable for the CHWs, was found to be an effective strategy to enhance performance, especially of those CHWs with multiple tasks. Performance-based financial incentives sometimes resulted in neglect of unpaid tasks. Supervision and training were often mentioned as facilitating factors. Embedment of CHWs in community and health systems was found to diminish workload and increase CHW credibility.
Auxiliary midwives in hard to reach rural areas of Myanmar: filling MCH gaps	Wangmo *et al.* (2016)	Auxiliary Midwives (AMWs) are community health volunteers supporting the work of midwives, especially maternal and child health services in hard to-reach areas in Myanmar.			AMWs were able to provide essential maternal and child health services.90 % of the respondents expressed receiving no adequate supervision, refresher training, replenishment of the AMW kits and transportation cost.
Unlocking community capability through promotion of self-help for health: experience from Chakaria, Bangladesh	Bhuiya *et al.* (2016)	One mechanism to promote participation in health is through participatory action research (PAR) methods.	People’s participation in health, enshrined in the 1978 Alma Ata declaration, seeks to tap into community capability for better health and empowerment.	ICDDR,B implemented a project “self-help for health,” to work with existing rural self-help organizations (SHOs). SHOs are organizations formed by villagers for their well-being through their own initiatives without external material help.	SHO functionality increased improved organizational processes and planned health activities, while decreases in infant mortality and increases in utilization of at least one antenatal care visit occurred similarly in intervention and comparison areas, increases in immunization, skilled birth attendance, facility deliveries and sanitary latrines were substantially more in intervention than comparison areas.
Citizen Participation and Political Accountability for Public Service Delivery in India: Remapping the World Bank’s Routes	Blair (2018)	A state’s accountability to its citizens for public service delivery constitutes a central component of the democratic polity.	The linkage between citizens and some combination of elected political leaders and those they direct to provide the services.	Explores the paths these three routes cantake and their potential effectiveness in providing citizens a number of institutionalmechanisms to hold political leaders and public service providers accountable,improve service delivery, empower poor people and ultimately enhancewell-being.	Civil societies can directly addressing the state bureaucracy in seeking changes in how a policy is implemented.

**Public hearings**					

Preventing Corruption in Public Service Delivery in Bangladesh	Ahmed (2016)	According to all major global indicators of corruption, Bangladesh is one of the most corrupt countries in the world as per Transparency International according to its Corruption Perception Index (CPI).	The Anti-Corruption Commission (ACC) conducts public hearings at the upazila level for ensuring the accountability of public officials and also transparency of their work.	This study is based on the written complaints raised by 1440 citizens in 72 public hearings conducted by the Anti-Corruption Commission (ACC) in Bangladesh.	TIB conducted a study of 13 public hearings with 195 respondents. The reasons for liking public hearings was that it created opportunities for making authorities accountable to citizens (75%) followed by the opportunity to raise complaints before officials (69%) and commitment to solve complaints (20%).
**Community Action Groups**					

Women in the lead monitoring health services, Naripokkho, Bangladesh	COPASAH (2015)	Naripokkho works in all the 64 districts of Bangladesh on empowerment and reproductive health and rights of women specially in rural setting.	To strengthen the accountability mechanism in the health service delivery mechanism among rural women.	NariDal a village health facility monitoring group by village women’s.	Helped to raise awareness on health rights and increase the use of health services in the village women by enhancing accountabilities in service provision at local health facilities.

**Participatory Complaints Survey**					

Local Governance: Accountable Public service in Indonesia	GTZ (2009)	Challenges of equal access to quality services due to vast geographical area.	Increasing the accountability of the public sector, improvement of public services through civil society participation.	Complaint survey conducted by service units.	Citizens were more aware and empowered, there was an improvement in service provision; service users also had less complaint than earlier with regards to constraints in service provision.

**Social Audits**					

Making local health services accountable. Social auditing in Nepal’s health sector.	GIZ, (2014)	Nepal at local level, public health facilities across the country face daunting problems, including insufficient supplies of drugs and basic equipment, understaffing and absenteeism, and low level of accountability to local people.	Social auditing has been introduced on an increasingly wide scale to enhance citizens’ ability to participate in decision making about their health services at facility level.	In 2013-14, a total of 602 facilities in 45 districts (i.e. the majority of the country’s 75 districts) held social audits.	Social audits increased demand for services by informing people. Staffing shortages were fully or partially filled. The challenge of drug stock-outs and infrastructure problems with buildings and equipment were effectively dealt. On a broader scale, the social audit added value in such as giving facility in-charges opportunities not just to respond to questions and concerns, but educate local community members,
The role of social audit as a social accountability mechanism for strengthening governance and service delivery in the primary health care setting of Nepal: a qualitative study	Gurung *et al.* (2019) Kumar (2019)	Social audit is a mechanism used to hold frontline health service providers accountable.	Using the case of the social audit process in Dang District, Nepal, this study explored the role of social audit in facilitating direct accountability between service providers and community.	A total of 39 interviews were held with health facility operation and management committee members, service providers, district level health managers and non-government organization members. Reviews of records of social audit action plans were undertaken at 10 health facilities.	Participants reported that the social audit process was able to facilitate information provision/data collection, and provided opportunities for dialogue between community and service providers, but the provision of sanctions was found to be weak. While social audit had a positive role in increasing transparency, accessibility and quality of services, its effectiveness in addressing perennial governance problems was mixed. Manipulation of the participation process, falsification of information, and lack of authority affected the role of social audit in facilitating accountability.
Anti-corruption Measures in India: A Democratic Assessment		Social Audit is a tool with which government departments can plan, manage and measure non-financial activities and monitor both internal and external consequences of the department/organization’s social and commercial operations. It is an instrument of social accountability for an organization.	Assesses the impact of anti-corruption measures adopted in India since independence and seeks to find out why, despite a robust anti-corruption framework, these measures have failed to tackle corruption in the country.	A comparative review of different anti-corruption measures adopted in different countries done.	The bureaucratic nature of the audit team, lack of awareness among the people and minimum focus on social mobilization have been some of the main reasons for the ineffectiveness of social audit in India. Thus, the practical problem with social audit is that it is only concerned about the outputs of government policies and not about the outcomes. In other words it is mainly concerned with the numbers that are present on paper and not on the results, performance or actual achievements

**Health Facility Management**					

Fostering good governance at peripheral public health facilities: an experience from Nepal.	Gurung and Tuladhar (2013)	To foster good governance in the health facilities by increasing the capacity of HFOMCs.	To make this local committee responsible for managing all affairs of the health facility.	Health Facility Operation and Management Committees (HFOMCs).	Health Facility Management Strengthening Programme was quite successful in strengthening local health governance in the health facilities. The level of community engagement in governance improved.

The results are presented thematically below.

#### Local governance

Local governance was a social accountability approach to enhance transparency and accountability at the local level. In three studies (Cleary *et al.*, 2013; Garimella and Sheikh, 2016; Panda *et al.*, 2016), it was found to be effective by enabling space for decision-making at the local level. It improved health units’ performance by enhancing local authorities’ decision-making and enabling them to apply a bottom-up approach. Ensuring accountability was identified as a major role of local governance. A case study conducted in Nepal argued that local government accountability should be envisioned as an ongoing process and should be accompanied by systems of local planning and implementation, and revitalization of local democracy (Dhungana, 2019).

Evidence indicates that health system performance in achieving the objectives of efficiency, quality and equity is contingent on the breadth of ‘decision space’ at the local level. The functional areas of finance, service autonomy, recruitment rules, access rules and departmental rules normally have a very narrow ‘decision space’ at the local level, constraining the power of local authorities. In Odisha, India, Rogi Kalyan Samitis (RKS), a composite body of decision-making in peripheral health units, was formed with a mandate to ensure transparency in health facilities governance. For public service delivery health institutions, such as hospitals and healthcare centres, RKS was formed as an institution of local decision-making to take the public health system agenda forward. The functions of RKS included: (1) governance (accountability, responsiveness and transparency); (2) infrastructure (construction and maintenance, purchase and out-sourcing); (3) human resources management (hiring, transfer and training of staff); (4) financial resource management (cost-cutting measures, resource generation); and (5) quality improvement (supervision, modernization, quality assurance and accreditation). Panda *et al.* conducted a study on RSK staff satisfaction which showed that the majority (87%) of respondents were ‘satisfied’ with their current roles. Almost all (98%) noted that local decision-making helped to improve the performance of health units (Panda *et al.*, 2016).

Garimella and Sheikh conducted a case study to explore posting and transfer at the primary level in Tamil Nadu, India, in the context of the complex governance system of the government health sector. The study emphasized the need for bottom-up approaches to address the complexity within the governance context. Moreover, the blurring boundaries between public–private actors needed to be addressed for coordinated efforts towards local governance interventions (Garimella and Sheikh, 2016). Cleary *et al.* examined accountability in district-level health system governance, and found it important to limit the potential negative impacts of powerful actors to leverage a shift towards well-functioning accountability. A balance between achieving accountability and allowing local-level innovation was suggested as helpful. Findings show that accountability mechanisms could be key tools for ensuring the answerability of public primary healthcare facilities to central bureaucracies through the district health system, while at the same time providing the local decision space that could increase citizen and patient responsiveness (Cleary *et al.*, 2013).

#### Citizen’s Charters

Citizen’s Charters are one new social accountability approach to inform citizens about service entitlements, standards and rights. A Citizen’s Charter is a document articulating the commitment of government organizations towards citizens through clearly specified measures (Sharma, 2012). In Bangladesh, introducing and enforcing a Citizen’s Charter has been seen to ensure transparency and integrity in large-scale public procurement (Shohag, 2018). However, Citizen’s Charters have been largely ineffective and have failed to have an impact on enhancing accountability due to the top-down approach of their implementation.

Citizen’s Charters are intended to provide information to citizens on the choice and standards of services that should be provided by an institution. During 2004, a Citizen’s Charter initiative in Chandigarh, India was adopted with the aim of improving public service delivery. The Charter was supposed to inform citizens about specific complaint centres. The 32-page document was divided into sections. One of its special features was the universal email address for all types of complaints. A case study by Sharma reveals that the Citizen’s Charter made little impact as it was not displayed anywhere accessible to citizens. Even the employees of the government agencies were not well informed. The top-down approach resulted in poor design, a lack of awareness or interest among stakeholders, a lack of information and an absence of an implementation strategy or community awareness and participation (Sharma, 2012).

In 2007, a Citizen’s Charter was introduced in Bangladesh to improve the delivery of quality services and of transparency and accountability at the local level. Huque and Ahsan conducted a survey in Rajshahi district to evaluate its impact. Findings revealed that only a small number of people were aware of its existence. Information was presented in a way that did not allow citizens to play a meaningful role. Respondents had no stake in the preparation or implementation of the Charter. The survey revealed a number of factors limiting the success of the initiative, which includes similar factors as for Chandigarh. It found that a top-down approach to adopting and implementing the Charter in a haphazard fashion may have contributed to its limited success (Sharma, 2012; Huque and Ahsan, 2016).

#### Social audits

Social audits are an asocial accountability tool to enhance service delivery transparency and accountability by improving participation (GIZ, 2014). In theory, social audits provide opportunities for mutual accountability by evaluating health system performance via citizens. The literature suggests that they can be effective for enhancing community roles in local healthcare service monitoring by raising service demand and enabling organizational change. Social audits are based on the idea that people’s participation in policy processes can become an effective tool to fight corruption, and that this can be achieved when people are aware of the nature and effects of corruption. Civil society groups in India have played a prominent role in raising awareness among citizens about the negative impact of corruption, which has also helped to strengthen government–citizen relations (Kumar, 2019). A social audit initiative in Andhra Pradesh state in India involved poor citizens. But the programme has been found ineffective in redressing and sanctioning functions; while it proved reasonably effective in detecting malfeasance, it did little to reduce it over three successive iterations (Blair, 2018).

Nepal has a long history of utilising social audits. From 2013 to 2014, its government conducted social audits in 602 facilities in 45 districts (out of a total 75 districts) with support from local and international development agencies (e.g. GIZ and the UN) to enhance community participation in decision-making and monitor local healthcare services. In this process, a Social Audit Committee was formed within districts. To disseminate findings within communities, a mass public gathering was organized ensuring the presence of facility service providers and authorities. Finally, a local action plan was developed to enhance transparency and good governance in facilities by assigning responsibilities. Vacant positions were also filled through temporary contracts, the behaviour of health workers improved, facilities were made more responsive to patients’ needs and the Health Facility Management Committees were reformed or re-energized. While findings about the impact were only tracked in two facilities, limiting the potential to learn from this intervention, it appears that the use of services increased, staffing shortages were fully or partially filled and drug shortage and infrastructure problems were solved (GIZ, 2014). Social audits have been used as a mechanism to hold frontline health service providers to account—e.g. the audit process in Dang District, Nepal facilitated direct accountability between service providers and the community. Participants reported that the process improved information provision and provided opportunities for dialogue between the community and service providers. While social audits have a positive role in increasing the transparency, accessibility and quality of services, their effectiveness in addressing perennial governance problems has been mixed. Manipulation of the participation process, falsification of information and communities’ lack of power have all affected the role of social audits in facilitating accountability. The study authors argued that it is essential to consider these factors while designing and implementing social audit processes and accountability mechanisms between service providers and the community (Gurung *et al.*, 2019). In Nepal, social audits have also been implemented to enhance accountability in maternal health services, but impacts on governance were mainly found at the local level. Factors contributing to the lack of broader impact were the absence of a mandate for community health volunteers to play an active role in the social accountability process, and limited capacity, including of resources (Hamal *et al.*, 2019).

#### Score cards

Score cards are a quantitative approach typically involving surveys of citizen satisfaction, which include a facilitated meeting between providers and beneficiaries to discuss results and agree on follow-up actions (World Bank Group, 2012). Work on community scorecards is intended to be a participatory, community-based social accountability approach to evaluate and improve public services, and to inform and empower local actors. The use of score cards was found to be effective for monitoring quality in service provision in one study. A facility survey using a score card conducted in Bangladesh reported it as useful to better understand various aspects of service delivery through gathered data, which also helped to strengthen the management information system itself (Khan *et al.*, 2013). In Myanmar, the 3MDG Fund trained its implementing partners (IPs) on the use of community score cards as a participatory approach for communities and service providers to engage in dialogue on the delivery of services (3MDG, 2016).

A score card health survey was conducted in 80 health complexes (upazila) in Bangladesh in 2009. A list of basic medical equipment was used to calculate equipment availability for health facilities. More than 60% of the facilities were found to have at least 75% of the basic medical equipment. In terms of human resources, both physicians and nurses were in short supply at all levels of the healthcare system. The overall job satisfaction index was <50 for physicians and 66 for nurses out of 100, with 100 being very satisfied. The score card approach was found useful for monitoring quality (Khan *et al.*, 2013).

#### Participatory complaints surveys

Participatory complaints surveys are a participatory social accountability tool to enhance service provision accountability. Complaints surveys empower citizens to hold authorities accountable for given services. They are effective for identifying gaps in service provision, and can improve local-level planning.

As a vast country with a population of over 240 million, spread out over about 6000 inhabited islands, Indonesia faces enormous challenges with the provision of equal access to quality services. Since 2000, the State Ministry of Administrative Reform has implemented a Support for Good Governance initiative. A representative patient complaint survey was developed through workshops with service users (80%) and service unit staff (20%) led by trained facilitators. A complaint survey was conducted at 60 service units with a minimum of 80% of service users at the lowest level of service provision. Typical complaints about the services of Local Health Centres (PusKesMas) included lack of medical personnel, lack of discipline/skills/information sharing of medical personnel, lack of medication, variety in pricing and finally exorbitant costs for medication. A number of districts and municipalities (74 out of approximately 500) applied this participatory method, reacting to the complaints of 380 000 respondents. A number of districts and municipalities continue to expand this approach into new sectors, often at their own cost. A few service units at the provincial and national level (the customs bureau) have also successfully applied the method. As patient compliant surveys were repeated, citizens’ became more aware and empowered, and there was an improvement in service provision; service users also had fewer complaints regarding service provision (GTZ, 2009).

#### Community volunteer/participation

Community participation is defined as the involvement of people in a community in projects to solve their own challenges (World Bank Group, 2012). Community participation in the form of community volunteers or health workers was found to be effective for enhancing system performance in resource constraint scenarios. It was also effective in bridging the community and systems by identifying community needs. It positively impacted local resource mobilization and helped organize activity planning.

Community health workers (CHWs) are an effective way to enhance performance in preventive, curative and promotional primary healthcare services in LMICs with a given mix of financial and non-financial incentives (Kok *et al.*, 2015; Wangmo *et al.*, 2016). A systematic review of 140 quantitative and qualitative studies identified factors related to the nature of tasks and time spent on delivery, human resource management, quality assurance, links with the community, links with the health system and resources and logistics having an influence on CHW performance. Good performance was associated with intervention designs involving a mix of incentives, frequent supervision, continuous training, community involvement and strong coordination and communication between CHWs and health professionals, leading to the increased credibility of CHWs (Kok *et al.*, 2015).

Myanmar faced critical resource constraints, creating major gaps in access to and coverage of health services. Recognizing the benefits of community-based health workers, Myanmar trained, deployed and integrated auxiliary midwives (AMWs) in the health system, to deliver maternal and child health services (MCH) services to hard-to-reach and remote areas. A quantitative cross-sectional study was conducted in 2013 to assess the extent of AMWs’ contribution to addressing the shortage of midwives. AMWs were able to provide essential maternal and child health services including antenatal care, normal delivery and post-natal care, and had a comparative advantage due to longer service in hard-to-reach villages where they lived, speaking the same dialect as the locals, understanding the socio-cultural dimensions and being well accepted by the community. Despite these contributions, challenges remain; e.g. 90% of AMWs stated that they had received no adequate supervision, refresher training, replenishment of AMW kits or reimbursement of transportation costs (Wangmo *et al.*, 2016).

In 2005, in India, the National Rural Health Mission launched to revamp the rural health system. An ethnographic study was conducted in Odisha to gather evidence on community interaction. It showed that for health workers, the notion of integration goes well beyond a technical lens of mixing different health services. Crucially, ‘teamwork’ and ‘building trust with the community’ (beyond trust in health services) are critical components. Evidence shows that highly hierarchical health bureaucratic structures, which rest on top-down communications, limit efforts towards sustainable health system integration (Mishra, 2014).

Unlocking community capabilities through self-help organizations (SHOs) was helpful for bridging between the community and the system. An international research organization in Bangladesh, ‘ICDDR, B’, implemented a project on ‘self-help for health’ to work with existing rural SHOs. SHOs are organizations formed by villagers for their well-being through their own initiatives without external material help. Following a self-help conceptual framework, the project focused on building the capacity of SHOs and their members through training on organizational issues, imparting health literacy and supporting participatory planning and monitoring. Villagers and members of the SHOs actively participated in the self-help activities. SHO functionality increased in the intervention area, in terms of improved organizational processes and planned health activities. These included convening more regular meetings, identifying community needs, developing and implementing action plans and monitoring progress and impact. Between 1999 and 2015, while decreases in infant mortality and increases in utilization of at least one antenatal care visit occurred, increases in immunization, skilled birth attendance, facility deliveries and sanitary latrines were substantially higher in the intervention area than in the comparison area (Bhuiya *et al.*, 2016).

In Jhenaidah, Bangladesh, a community-driven initiative helped to mobilise 46 additional workers from the local community for the Chowgacha health complex (sub-district health facility) by involving communities, particularly including locally influential individuals (SHARE, 2016). The 3MDG fund is working with existing IPs to strengthen their engagement with communities, including poor and vulnerable populations, by providing support to improve their approach to participation, inclusion, information sharing and responding to community feedback in Myanmar (3MDG, 2012). Community participation helped to mobilize local resources and to raise collective demands through community empowerment (3MDG, 2012) as well. It needed the active involvement of citizens, as well as the facilitation, communication and a change in mindset of the administrators. A high turnover and a lack of incentive limited community-led interventions. To practice social accountability approach understanding of the local context and perspective of both community and provider, building community capacity and ownership, linking the interventions to the formal system and meeting community demand were all found to be crucial for such interventions (3MDG, 2016; Bhuiya *et al.*, 2016; Wangmo *et al.*, 2016).

#### Community action groups

Community action groups (CAGs) are another form of community-led social accountability found to be effective in ensuring the inclusion of marginalized communities. They can raise collective voices and increase service demand, especially among poor women.

Experiments with community-led approaches have been practised to raise collective voices in this region (Asia Pacific Network, 2011; COPASAH, 2015; Bhuiya *et al.*, 2016a). For example, ‘Naripokkho’ in Bangladesh is a national membership organization working on women’s rights since 1983 to empower women (COPASAH, 2015). To strengthen accountability mechanisms in 2003, Naripokkho initiated a ‘Women’s Health Right’s Advocacy Partnership’ (WHRAP) in five districts with 16 NGOs and 640 active members. Under this WHRAP initiative, marginalized women were organized into groups named ‘NariDal’ in villages to monitor health services. As the poor and marginalized women of the community are reluctant to use available healthcare services, the NariDal members advocate with marginalized women for their health rights. In these meetings, issues like availability of healthcare services (e.g. facilities, medicines), women’s health conditions, health rights, entitlements and obligations of providers are discussed. Though the NariDal members faced challenges from within the family and community for their involvement, through regular meetings they raised awareness on health rights and increased the use of health services by women in villages by enhancing accountability in service provision (COPASAH, 2015).

Between 2009 and 2011, Bhutan established CAGs in four districts (Asia Pacific Network, 2011). The group members included local government representatives, village health workers, religious group members and representatives from different sectors ensuring female representation. The group discussed priorities and developed a local action plan. They met quarterly and sent reports to the central level every six months. The initiative was reported as helpful and having improved village sanitation. Although the high turnover of village health workers was a major challenge, this approach was helpful to create community ownership of health activities, stimulate decentralization and build capacity of local leadership. CAG members receive a three-day training course covering sanitation, community motivation, nutrition and child care. CAGs were successful in improving sanitation in the villages (Asia Pacific Network, 2011).

#### Public hearings

Public hearings are defined as formal meetings at the community level where citizens express their grievances on matters of public interest to public officials who try to address these grievances (Ahmed, 2016). Public hearings are expected to provide a platform through which citizens can call authorities to account. They were found to be effective for exposing corruption and mismanagement in public service provision, but there is limited evidence on their impact (Ahmed, 2016).

Public hearings have been practised as a means of empowering citizens with information on public services and raising collective voices. This involves public officials and citizens of the same locality, and allows citizens to question the authorities directly on irregularities in public services. The Anti-Corruption Commission in Bangladesh organized 72 public hearings by 1440 citizens in 61 upazilas of 51 districts and in two metropolitan cities up until 2017. As per the public hearing findings, systematic corruption prevails in public service delivery and health was identified as one of the most corrupt service departments. An absence of citizen engagement was mentioned as a reason behind the corrupt practices (Ahmed, 2016). A follow-up survey conducted by TI, Bangladesh in 2017 found that 75% of respondents liked public hearings as a platform to make authorities accountable to citizens. Sixty-nine per cent thought that public hearings provide the opportunity to raise complaints before officials. The study also revealed that as a result of holding public hearings, authorities have taken measures like putting out more information boards, providing complaint boxes, improving filing systems and monitoring through CCTV to improve public service delivery (Ahmed, 2016). The steps taken after public hearings demonstrate that these mechanisms appear to be effective instruments in corruption prevention, this is not supported by the evidence.

#### ‘Ayauskam’: A classic case of practising different social accountability tools

The ‘Ayauskam’ project in India led by a civil society organization is a classic example where different social accountability tools were used to reduce corruption and improve service delivery responsiveness (PTF, 2012). Initiated in 1993 in the State of Odissa, Ayauskam conducted a baseline survey in 64 villages to explore corruption. It established community-based organizations and organized public hearings. It also organized a broad campaign against corruption, holding rallies and demonstrations and using media to protest corruption. Ayauskam faced several challenges throughout the process. The service providers, government officials and local politically influential people were not supportive, and obstructed efforts. They even filed criminal cases and made false claims against Ayauskam. The project staff made frequent efforts to have discussions with the authorities, service providers and local influential people. Through gradual cooperation between the them, Ayauskam was able to make it clear that it was combating corruption and not individuals. The authorities recognized the strength of community and thus started initiatives like village health committees, which increased community participation in the decision-making and monitoring processes. The intervention helped to improve child nutrition and antenatal and postnatal services. There was a reduction in corruption practices in government hospitals in the project area, with 80% of those surveyed not needing to pay a bribe for giving birth at local hospitals (PTF, 2012).

#### Broader reforms to enable social accountability

In addition to the above initiatives, our review identified evidence on social accountability as a part of comprehensive health system reform packages—presented in the ‘Good governance’ and ‘Decentralization’ sections of this article. In many of these, there were implicit measures to involve end users and citizens and to ensure feedback loops and responsiveness.

#### Good governance

Good governance, defined by Huss *et al.* (2011) as the exercise of power through institutions to steer society for the public good, has been practised as a model to enhance transparency and accountability within systems. Indonesia established an anti-fraud system within its universal health insurance, and a study sought to analyse its operation through a good governance lens. Findings indicate that good governance principles are essential in designing an effective anti-fraud system due to the correlation between human rights and anti-corruption; both areas emphasize good governance principles as fundamental for the realization of human rights and the making of a viable anti-corruption strategy (Juwita, 2018). Good governance approaches were also aimed at enhancing accountability and improving service delivery in Karnataka, India (Huss *et al.*, 2011). A public complaint agency (KLA) was created in Karnataka state in India in 1986, which played a prominent role in controlling systemic corruption. KLA had the authority to investigate complaints from citizens about public maladministration and to initiate prosecution for criminal offences. In the initial phase, KLA was criticized by the Karnataka High Court and Karnataka Administrative Reform Commission for its failure to hold governments accountable, ensure effective redressal of grievances and improve public administration governance. Later, the post of Vigilance Director for Health, Education and Family Welfare (VDH) was created and under strong leadership it became widely known, gaining a reputation for independence and a strong will to fight maladministration. Thus, the change in leadership in 2001 and the creation of the position of Vigilance Director for Health improved the effectiveness of the KLA. The Karnataka experience showed that a shift towards good governance requires the interaction of leaders, followers and system changes. An effective accountability mechanism requires a committed and powerful leadership, adequate resources, robust capability to investigate and deal with internal governance issues and the authority to propose institutional reforms (Huss *et al.*, 2011).

#### Decentralization

Decentralization is defined as a socio-political process of power-sharing arrangements between central government and local authorities in planning, management and decision-making (Regmi *et al.*, 2010). Decentralization was aimed at enhancing accountability with resources via power transformation at the local level. It was effective in terms of bringing services close to citizens.

Decentralization has had a positive impact in improving district health service provision, especially in planning and management with a clear local agenda. For example, within the decentralization framework of government, the Ministry of Health (MoH) Nepal initiated the decentralization of primary care services closer to citizens in 1999. Regmi *et al.*’s (2010a) study on decentralization revealed that service users considered decentralization as a means of transferring authorities and accountabilities with resources (both human resource and finance) from the centre to local authorities and community healthcare facilities. This was viewed by respondents as a possible advantage of decentralization for the local government. Decentralization was positively associated with increased service access and utilization and improved service delivery. Most of the districts’ health service facilities (health institutions) were handed over to local committees. The main purpose of restructuring was to involve community people and bring health services closer to citizens. The study reported decentralization impacting positively on the district health services in terms of service provision, community participation and empowerment, service planning, management and coordination. It also identified the barriers to implementation, such as difficulties in developing capacity, monitoring and accountability systems, clarity in roles and responsibilities and also fund allocation and distribution (Regmi *et al.*, 2010a).

In Nepal, decentralized human resource management was practised by handing over the health facilities of 28 districts to local bodies (Devkota *et al.*, 2013; Gurung and Tuladhar, 2013) and forming village development and district health development committees. The initiative promoted ownership at the local level as a result of resource sharing to equip health facilities, and to improve recruitment and retention of staff locally. However, weak monitoring has been seen as a key obstacle in promoting local leadership (Devkota *et al.*, 2013). Nepal’s experience revealed that successful implementation of decentralization requires a broader context of institutional capacity building and resource management and underlines the need for consideration of these during implementation processes (Regmi *et al.*, 2010b). The active involvement of service users, providers and policymakers in the process of decentralization and clear local level agendas were reported crucial for such initiatives (Regmi *et al.*, 2010b). The effects of decentralization on corruption are mixed, and the types, dynamics of corruption and impact of different anti-corruption approaches vary in different decentralized contexts. Conflicting provisions regarding administrative authority at the different levels affect the effective functioning of local-level institutions and their ability to govern, and this requires strengthening of the upward and downward accountability mechanisms (Bhattacharya *et al.*, 2018).

## Discussion

This review of the literature on social accountability and its impact on governance in the health sector sought to explore alternative, bottom-up and community-engaged interventions to improve governance and combat health sector corruption in selected LMICs in South and Southeast Asia. Findings reveal a multitude of experiments in different countries of the region to strengthen health sector governance through ‘social accountability’ initiatives. Our review demonstrates that different countries (e.g. Bangladesh, Bhutan, India, Indonesia, Myanmar, the Maldives and Nepal) have implemented a broad variety of social accountability mechanisms, e.g. social audits, score cards, participatory complaints surveys, public hearings, community volunteers and community actions groups.

Key themes emerging from the review were: transparency, accountability and community participation. Across the papers on transparency approaches, strong political commitment, appropriate policy design and active participation of citizens appeared to be key to effective implementation of such interventions; however, the link between these factors and the success of social accountability programmes is only tentative. Similarly, the absence of these factors undermined the application of the approaches based on transparency. Transparency intervention such as Citizen’s Charters often failed due to poor design following a top-down approach, poor political commitment and lack of citizens’ involvement through a participatory process. Approaches based on strengthening local governance, on the other hand, allowing space for decision-making at the local level, were found to have potential provided there was strong leadership. However, the balance between monitoring accountability and allowing a local-level decision-making space was a challenge to such interventions. Thus, the evidence on whether incorporating these key elements into the interventions can ensure an impact is inconclusive.

Across the papers reviewed under accountability approaches, we found that effective design and implementation capacity were considered crucial for interventions like social audits, participatory complaint surveys and score cards, while the absence of robust guidelines or skilled facilitators of the process were constraining factors. Moreover, the active participation of citizens and mechanisms to follow up on complaints were a clear prerequisite. There was reasonable evidence that the interventions were effective in enhancing local-level accountability, monitoring service quality and empowering citizens through active involvement in shaping service provision. However, there was limited evidence that accountability approaches such as public hearings were effective beyond providing a platform to raise citizens’ voices. Evidence suggests that only coordinating citizens may fall short in achieving governance and service delivery improvements unless these processes are institutionalized and linked with systematic reform. Overall, the accountability approaches emphasized the mutual responsibility and participation of both service providers and communities, with similar findings reported in a social accountability study in Malawi (Gullo *et al.*, 2017).

Apart from specific accountability approaches, broader reforms like decentralization and good governance can play an important role in promoting social accountability, but they largely depend on institutional context and capacity. Governance is recognized as a cornerstone of a well-performing health system, and initiatives aimed to improve governance are especially important in less-developed countries whose health systems face numerous constraints (WHO, 2007). For this reason, a number of initiatives have sought to improve governance with a focus on strengthening legal frameworks, regulatory capacity and enforcement powers (Mikkelsen-Lopez I *et al.*, 2011). However, despite being effective at the individual programme level, evidence demonstrates that top-down approaches remain insufficiently effective and often create new areas for rule-breaking and governance failures (Khan *et al.*, 2019). The proliferation of social accountability (bottom-up) approaches takes a different perspective; it argues for involving and empowering relevant key stakeholders and a broad range of policymakers, ensuring that there are formal mechanisms to channel their concerns into actions and achieve changes in the health system. Khan *et al.* (2019) argue that, by aligning incentives and motivating at least some powerful sectoral organizations, sectoral strategies can be applied by organizations to address sector-specific problems.

Across the papers reviewed under community participation interventions, we found that participation in the form of community volunteer/worker/action groups is common in many social accountability programmes. Understanding and building on the perspective of both communities and providers and creating community ownership through identifying roles were a prerequisite of community-led interventions. The papers reviewed suggest that these approaches helped in increasing healthcare demand by raising collective voices and ensuring the inclusion of marginalized communities. However, the success of participation-based strategies depends critically on local power relations and on citizens’ capacity for collective voice. Thus, some of the interventions remain effective to an extent, achieving their intermediate goals by establishing inclusive and participatory processes; however, evidence on how these can be scaled up to a broader population and sustained, or how they can improve substantively health outcomes, is limited.

Most of the papers reviewed the technical preconditions for implementing effective social accountability models. Many of these are based on good intentions but may underestimate the importance of strategic design—not only of the interventions per se but also of their fit into the overall health system. As highlighted in the last section, social accountability initiatives are often part of packages of managerial interventions to counter the effects of poor governance—e.g. to improve human resources management and increase transparency. Another set of papers showed that broader decentralization reforms were mainly aimed at improving efficiency in quality service delivery and enabling local autonomy in decision-making; these were not clearly linked to social accountability approaches. Therefore, an important lesson may be that social (bottom-up) accountability has to be implemented in conjunction with top-down approaches (e.g. potentially using co-design and co-production) (Manikam*et al.* 2017; McAuliffe *et al.*, 2017; Beran *et al.*, 2018; Ward *et al.*, 2018). Implementing social accountability as a stand-alone intervention may be ineffective if institutional and contextual factors are opposing it (UNDP, 2013). Thus, the review showed that creating spaces for decision-making at the local level was useful but was constrained by administrative needs, capacity and other contextual factors.

Another critical point that is little discussed in the literature is the role of power (Shiffman, 2014). The review showed that the citizens and their communities played a central role in the accountability mechanisms, giving them voice via awareness building and provision of information, and this was frequently practised for each of the social accountability mechanisms, from the local to the national level. While many of the papers explicitly acknowledge that support from powerful actors was a necessary precondition for implementing the interventions (Hickey and King, 2016), the underlying premise––that the intervention is often designed and implemented in a way that may not challenge or that may even enhance the underlying power structures—was not explicitly examined.

## Conclusion

This review synthesized a diverse type of social accountability initiatives implemented in selected countries in South and Southeast Asia to enhance transparency, accountability and citizens’ participation. The initiatives and interventions differed from context to context, were initiated by a range of actors and occurred at various levels. Findings indicated that success (perceived or measured) largely depended on context, capacity, information, spectrum of actor involvement, independence from power agendas and leadership. Overall in different contexts, social accountability mechanisms are reported to have enhanced efficiency in service delivery, increase responsiveness and establish and strengthen links between citizens and the system. However, there is no common blueprint to ensure accountability mechanisms are viable and effective. Therefore, conclusions should be cautious as many of the papers do not reliably assess the outcomes of social accountability interventions (final or intermediate) using sufficiently rigorous qualitative or quantitative methods, often focusing on the process indicators or observations of those involved in these.

## Policy implications

The learning from the bottom-up accountability approaches reviewed in this study suggests that they are promising and often lead to positive effects locally; however, institutional and policy support can be important for implementing the approaches in a sustainable manner. This is especially helpful when the traditional ‘carrot and stick’ approaches to contain poor governance and corruption in the health sector appear to be ineffective and inconsequential, and instead more conciliatory and participatory approaches involving multiple stakeholders are considered.

## Strengths and limitations of the study

The review provides comprehensive information on the processes and experiences of social accountability interventions practised in selected countries in Southeast Asia. Strengths include the approach adapted to searching the literature, which included published peer-reviewed articles and grey materials and included a broad range of study designs, allowing us to provide a detailed map of the evidence on governance and social accountability. While offering insights by identifying key themes in this research area, it highlights the need for a full systematic literature review to arrive at conclusive findings. The review was restricted to English language documents only, and only three search engines were used. No formal quality assessment of the included sources was conducted. However, three researchers screened the papers and materials for appropriateness to the research question, working individually and then in a group, to review and synthesize data, maintaining consistent application of the inclusion and exclusion criteria. This article mainly focused on presenting innovative approaches practised, but not all articles provided sufficient information to report. Resource constraints prevented us from conducting a wider search, especially covering country-specific literature produced by non-academic and civil society stakeholders at the country level, which would undoubtedly be relevant. However, the review enabled us to explore a broad range of innovative approaches practised for improving governance and accountability in health systems, which have relevance to LMICs worldwide.

## References

[czaa107-B1] 3MDG. 2012 *Progress Report. Pact Building Local Promise* www.pactworld.org https://www.3mdg.org/en/engaging-communities, accessed 1 October 2020.

[czaa107-B2] 3MDG. 2016 *Community Scorecards. Linking Communities with Service Providers to Improve Service* https://themimu.info/sites/themimu.info/files/assessment_file_attachments/report_community_scorecard_workshop.pdf, accessed 1 October 2020.

[czaa107-B3] Ackerman J. 2004 Co-governance for accountability: beyond “exit” and “voice”. World Development 32(3): 447–63. DOI: 10.1016/j.worlddev.2003.06.015, accessed 1 October 2020.

[czaa107-B4] Ahmed N. 2016 *Preventing Corruption in Public Service Delivery in Bangladesh* Available at:https://bea-bd.org/site/images/pdf/new17/2.pdf, accessed 1 October 2020.

[czaa107-B5] Asia Pacific Network. 2011 *Bhutan: Community Action Groups—Building Local Participation for Improvement in Public Health* Available at:http://malariamatters.org/bhutan-community-action-groups-building-local-participation-for-improvement-in-public-health/, accessed 1 October 2020.

[czaa107-B6] Beran D, Lazo-Porras M, Cardenas MK et al 2018 Moving from formative research to co-creation of interventions: insights from a community health system project in Mozambique, Nepal and Peru. BMC Global Health 3(6):1–8. Available at: gh.bmj.com/content/3/6/e001183, accessed 1 October 2020. 10.1136/bmjgh-2018-001183 PMC6254743 30498592

[czaa107-B7] Bhattacharya D, Rezbana US and Fuad SM. 2018 Decentralised Governance, Corruption and Anti-corruption Measures: An Enquiry in Bangladesh Experience. Centre for Policy Dialogue (CPD) Project Report. Available at: https://cpd.org.bd/wp-content/uploads/2018/05/CPD-ODI-Project-Report-Decentralised-Governance-Corruption-and-Anti-corruption-Measures-An-Enquiry-in-Bangladesh-Experience.pdf, accessed 1 October 2020.

[czaa107-B8] Bhuiya A, Hanifi SMA, Hoque S et al 2016 Unlocking community capability through promotion of self-help for health: experience from Chakaria, Bangladesh. BMC Health Services Research 16(624): 105–17. Available at: bmchealthservres.biomedcentral.com/articles/10.1186, accessed 1 October 2020. 28185584 10.1186/s12913-016-1865-9 PMC5123251

[czaa107-B9] Blair H. 2018 Citizen participation and political accountability for public service delivery in India: remapping the World Bank’s routes. Journal of South Asian Development 13: 54–81.

[czaa107-B10] Boydell V. 2018 Why social accountability in health matters: the growing evidence, special thematic issue. COPASH. Special Thematic Issue 22: 3-8. Available at: copasah.net/uploads/1/2/6/4/12642634/newsletter_22pdf, accessed 1 October 2020.

[czaa107-B11] Boydell V, Kissbury J. 2014 *Social Accountability: What Are the Lessons for Improving Family Planning and Reproductive Health Programs?* A Review of the Literature. Working Paper. Washington, DC: Population Council, Evidence Project.

[czaa107-B12] Brinkerhoff DW. 2004 Accountability and health systems: toward conceptual clarity and policy relevance. Health Policy and Planning 19: 371–9. 15459162 10.1093/heapol/czh052

[czaa107-B13] Bruen C, Brugha R, Kageni A et al 2014 A concept in flux: questioning accountability in the context of global health cooperation. Globalization and Health 10: 73. 25487705 10.1186/s12992-014-0073-9 PMC4258948

[czaa107-B14] Carmen M, Reiner F, Janmejay S. 2004 Social accountability: an introduction to the concept and emerging practice (English). *Social development papers no.* 76. Washington, DC: World Bank.

[czaa107-B15] Cleary SM, Molyneux S, Gilson L. 2013 Resources, attitudes and culture: an understanding of the factors that influence the functioning of accountability mechanisms in primary health care settings. BMC Health Services Research 13: 320. 23953492 10.1186/1472-6963-13-320 PMC3844434

[czaa107-B16] COPASAH. 2015 Women in the Lead Monitoring Health Services, Naripokkho, Bangladesh. *Series on social accountability* http://www.chsj.org/uploads/1/0/2/1/10215849/case_study_1.pdf, accessed 1 October 2020.

[czaa107-B17] Devkota B, Ghimire J, Devkota A et al 2013 Health governance at local level from human resource for health perspectives: the case of Nepal. Journal of Nepal Health Research Council 11: 133–7. Available at: pubmed.ncbi.nlm.gov/24362600/, accessed 1 October 2020. 24362600

[czaa107-B18] Dhungana HP. 2019 The prospect of accountability in local governance in Nepal. Journal of Management and Development Studies 29: 1–19.

[czaa107-B19] Dieleman M, Shaw DM, Zwanikken P. 2011 Improving the implementation of health workforce policies through governance: a review of case studies. Human Resources for Health 9: 10 (2011). DOI:https://doi.org/10.1186/1478-4491-9-10, accessed 1 October 2020. 21486438 10.1186/1478-4491-9-10 PMC3094272

[czaa107-B20] Gale NK, Heath G, Cameron E et al 2013 Using the framework method for the analysis of qualitative data in multi-disciplinary health research. BMC Medical Research Methodology 13: 117. 24047204 10.1186/1471-2288-13-117 PMC3848812

[czaa107-B21] Garimella S, Sheikh K. 2016 Health worker posting and transfer at primary level in Tamil Nadu: governance of a complex health system function. Journal of Family Medicine and Primary Care 5: 663. 10.4103/2249-4863.197310 PMC5290779 28217602

[czaa107-B22] George A. 2003 Accountability in health services: transforming relationships and contexts. Harvard Centre for Population and Development Studies 13(1):1–25. Available at: wgo.int/management/partnerships/accountability/healthservicespdf, accessed 1 October 2020.

[czaa107-B23] GIZ. 2014 *Making Local Health Services Accountable. Social Auditing in Nepal’s Health Sector* http://health.bmz.de/ghpc/casestudies/Making_local_health_services_accountable/index.html, accessed 1 October 2020.

[czaa107-B24] GTZ. 2009 *Local Governance: Accountable Public Service in Indonesia* Available at:www.institutfuermenschenrechte.de/uploads/tx_commerce/promising_practices, accessed 1 October 2020.

[czaa107-B25] Gullo S, Galavotti C, Sebert Kuhlmann A et al 2017 Effects of a social accountability approach, CARE’s Community Score Card, on reproductive health-related outcomes in Malawi: a cluster-randomized controlled evaluation. PLoS One 12: e0171316. 28187159 10.1371/journal.pone.0171316 PMC5302808

[czaa107-B26] Gurung G, Derrett S, Hill PC et al 2019 The role of social audit as a social accountability mechanism for strengthening governance and service delivery in the primary health care setting of Nepal: a qualitative study. Critical Public Health 1–12. DOI: 10.1080/09581596.2019.1667487.ISSN:0958-1596(Print) 1469-3682(online) journal homepag: https//www.tandfonline.com, accessed 1 October 2020.

[czaa107-B27] Gurung G, Tuladhar S. 2013 Fostering good governance at peripheral public health facilities: an experience from Nepal. Rural & Remote Health 13(2):2042. Available at: pubmed.ncbi.nlm.nih.gov/23528140/, accessed 1 October 2020. 23528140

[czaa107-B28] Hamal M, Heiter K, Schoenmakers L et al 2019 Social accountability in maternal health services in the far-western development region in Nepal: an exploratory study. International Journal of Health Policy and Management 8: 280–12. 31204444 10.15171/ijhpm.2019.05 PMC6571494

[czaa107-B29] Hickey S, King S. 2016 Understanding social accountability: politics, power and building new social contracts. The Journal of Development Studies 52: 1225–40.

[czaa107-B30] Huque SH, Ahsan K. 2016 Citizen’s charter and implementation failure: performance and local councils in Bangladesh. Public Administration and Policy 19.1: 6–22. Available at: www.researchgate.net/publication/304150345, accessed 1 October 2020.

[czaa107-B31] Huss R, Green A, Sudarshan H et al 2011 Good governance and corruption in the health sector: lessons from the Karnataka experience. Health Policy and Planning 26: 471–84. 21169338 10.1093/heapol/czq080

[czaa107-B32] Joshi A. 2013 Context Matters: A Causal Chain Approach to Unpacking Social Accountability Interventions. *Institute of Developmental Studies.* Egypt. https://globalizationandhealth.biomedcentral.com/articles/10.1186/s12992-014-0073-9, accessed 1 October 2020.

[czaa107-B33] Juwita R. 2018a. Good governance and anti-corruption: responsibility to protect universal health coverage in Indonesia. Hasanuddin Law Review 4(2):1–19. DOI:10.20956/halrev.v4i2.1424, accessed 1 October 2020.

[czaa107-B34] Juwita R. 2018b. Health sector corruption as the archenemy of universal health coverage in Indonesia. International Law and International Human Rights Departments. Faculty of Law Universitas Atma Jaya Yogyakarta.

[czaa107-B35] Khan M, Andreoni A, Roy P. 2019 Anti-Corruption in Adverse Contexts: A Strategic Approach. *ACE Working Paper.* SOAS University of London. https://ace.soas.ac.uk/wp-content/uploads/2019/12/ACE-BriefingPaper006-NG-191202.pdf, accessed 1 October 2020.

[czaa107-B36] Khan MM, Hotchkiss R, Dmytraczenko T et al 2013 Use of a Balanced Scorecard in strengthening health systems in developing countries: an analysis based on nationally representative Bangladesh Health Facility Survey. The International Journal of Health Planning and Management 28: 202–15. 22887590 10.1002/hpm.2136

[czaa107-B37] Kok MC, Dieleman M, Taegtmeyer M et al 2015 Which intervention design factors influence performance of community health workers in low-and middle-income countries? A systematic review. Health Policy and Planning 30(9): 1207–27. DOI: 10.1093/heapol/czu126, accessed 1 October 2020. 25500559 10.1093/heapol/czu126 PMC4597042

[czaa107-B38] Kumar P. 2019 Anti-corruption measures in India: a democratic assessment. Asian Journal of Public Affairs 11(2): 19.DOI:http://dx.doi.org/10.18003/ajpa.20191, accessed 1 October 2020.

[czaa107-B39] Lewis M. 2006 Governance and corruption in public health care systems. The Centre for Global Development. *Working Paper Number 78***.**

[czaa107-B40] Manikam L, Shah R, Reed K et al 2017 Using a co-production prioritization exercise involving South Asian children, young people and their families to identify health priorities requiring further research and public awareness. Health Expectations 20: 852–61. 27933711 10.1111/hex.12524 PMC5600270

[czaa107-B41] Mikkelsen-Lopez I, Wyss K, De Savigny D. 2011 An approach to addressing governance from a health system framework perspective. BMC International Health and Human Rights 11: 13. 22136318 10.1186/1472-698X-11-13 PMC3247022

[czaa107-B42] Mishra A. 2014 ‘ Trust and teamwork matter’: community health workers' experiences in integrated service delivery in India. Global Public Health 9: 960–74. 25025872 10.1080/17441692.2014.934877 PMC4166967

[czaa107-B43] McAuliffe E, De Brún A, Ward M et al 2017 Collective leadership and safety cultures (Co-Lead): protocol for a mixed methods pilot evaluation of the impact of a co-designed collective leadership intervention on team performance and safety culture in a hospital group in Ireland. BMJ Open 7: e017569. 10.1136/bmjopen-2017-017569 PMC5695301 29101137

[czaa107-B44] Naher N, Hassan MS, Hoque R et al 2018. Irregularities, informal practices, and the motivation of frontline healthcare providers in Bangladesh: current scenario and future perspectives towards achieving universal health coverage by 2030. ACE SOAS Consortium Working Paper 004.

[czaa107-B45] Panda B, Thakur HP. 2016 Decentralization and health system performance–a focused review of dimensions, difficulties, and derivatives in India. BMC Health Services Research 16: 561. 28185593 10.1186/s12913-016-1784-9 PMC5103245

[czaa107-B46] Panda B, Zodpey SP, Thakur HP. 2016 Local self-governance in health-a study of it’s functioning in Odisha. BMC Health Services Research 16: 554. 28185587 10.1186/s12913-016-1785-8 PMC5103239

[czaa107-B47] PTF. 2012 Strategies for Empowering Communities to Demand Good Governance and Seek Increased Effectiveness of Public Service Delivery. PTF Working Paper Series 4 Available at: ptfund.org/wp-content/uploads/2018/07/WP04-Empowering-Communities-to-Demand-Good-Governance.pdf, accessed 1 October 2020.

[czaa107-B48] Prasuna G, Kumar G. 2016 Social audit: current need in public health care sector. International Journal of Community Medicine and Public Health 3: 11–6.

[czaa107-B49] Regmi K, Naidoo J, Pilkington PA et al 2010a. Decentralization and district health services in Nepal: understanding the views of service users and service providers. Journal of Public Health 32(3): 406–17. DOI:10.1093/pubmed/fdp116, accessed 1 October 2020. 19995811 10.1093/pubmed/fdp116

[czaa107-B349] Regmi K, Naidoo J, Geer A et al 2010b. Understanding the effect of decentralisation of health services: The Nepalese experience. Journal of Health Organisation and Management 24: 361–82. 10.1108/14777261011064986 21033634

[czaa107-B50] Ringold et al 2012 *Citizens and Service Delivery: Assessing the Use of Social Accountability Approaches in Human Development* *Direction in Development: Human Development*. Washington, DC: The World Bank.

[czaa107-B51] Rosenbloom DH. 2017 Public Administration in South Asia: India, Bangladesh and Pakistan. A Comprehensive Publication Program. Washington: DC: Public Administration American University.

[czaa107-B52] SHARE. 2016 *Engaging People in Health Dialogue* http://blog.icddrb.org/2016/10/31/public-engagement-health-sector-icddrb-share/, accessed 1 October 2020.

[czaa107-B53] Sharma D. 2012 An evaluation of a citizen’s charter in local government: a case study of Chandigarh, India. Journal of Administration and Governance JOAAG 7(1): 86–95. Available at: joaag.com/uploads/7_1_7_Case_Sharma_Final_pdf, accessed 1 October 2020.

[czaa107-B54] Shiffman J. 2014 Knowledge, Moral Claims and the Exercise of Power in Global Health. Washington, DC: Department of Public Administration and Policy, American University. 10.15171/ijhpm.2014.120 PMC4226618 25396204

[czaa107-B55] Shohag MH. 2018 Corruption in the service sectors: revelation of a pragmatic explanation in context of Bangladesh. IOSR Journal of Humanities and Social Science (IOSR-JHSS) 23(8):46–61. Available at: www.iosrjournals.org[/iosr-jhss/papers, accessed 1 October 2020.

[czaa107-B56] Transparency International. 2009 *Transparency International Annual Report 2009* https://issuu.com/transparencyinternational/docs/annual report_web?mode=window&backgroundColor=%23222222, accessed 1 October 2020.

[czaa107-B57] UNDP. 2013 *Reflections on Social Accountability. Catalyzing Democratic Governance to Accelerate Progress towards the Millennium Development Goals* http://www.undp.org/content/dam/undp/documents/partners/civil_society/publications/2013 UNDP_Reflections-on-Social-Accountability_EN.pdf, accessed 1 October 2020.

[czaa107-B58] Vian T. 2007 Review of corruption in the health sector: theory, methods and interventions. Health Policy and Planning 23: 83–94. 10.1093/heapol/czm048 18281310

[czaa107-B59] Wangmo S, Suphanchaimat R, Htun WMM et al 2016 Auxiliary midwives in hard to reach rural areas of Myanmar: filling MCH gaps. BMC Public Health 16(1): 914. DOI:10.1186/s12889-016-3584-x, accessed 1 October 2020. 27586656 10.1186/s12889-016-3584-x PMC5007995

[czaa107-B60] Ward ME, De Brún A, Beirne D et al 2018 Using co-design to develop a collective leadership intervention for healthcare teams to improve safety culture. International Journal of Environmental Research and Public Health 15: 1182 10.3390/ijerph15061182 PMC6025638 29874883

[czaa107-B61] World Bank. 1992 Governance and Development. Washington, DC: World Bank.

[czaa107-B62] World Bank Group. 2004 World Development Report 2004: Making Services Work for Poor People. Washington, DC: World Bank Group.

[czaa107-B63] World Bank Group. 2012 Citizen and Service Delivery. Assessing the Use of Social Accountability Approaches in Human Development. Washington, DC: World Bank Group.

[czaa107-B64] World Health Organization. 2007 Everybody’s Business. Strengthening Health Systems to Improve Health Outcomes. WHO’S Framework for Action. Switzerland: WHO https://www.who.int/healthsystems/strategy/everybodys_business.pdf, accessed 30 October 2020.

